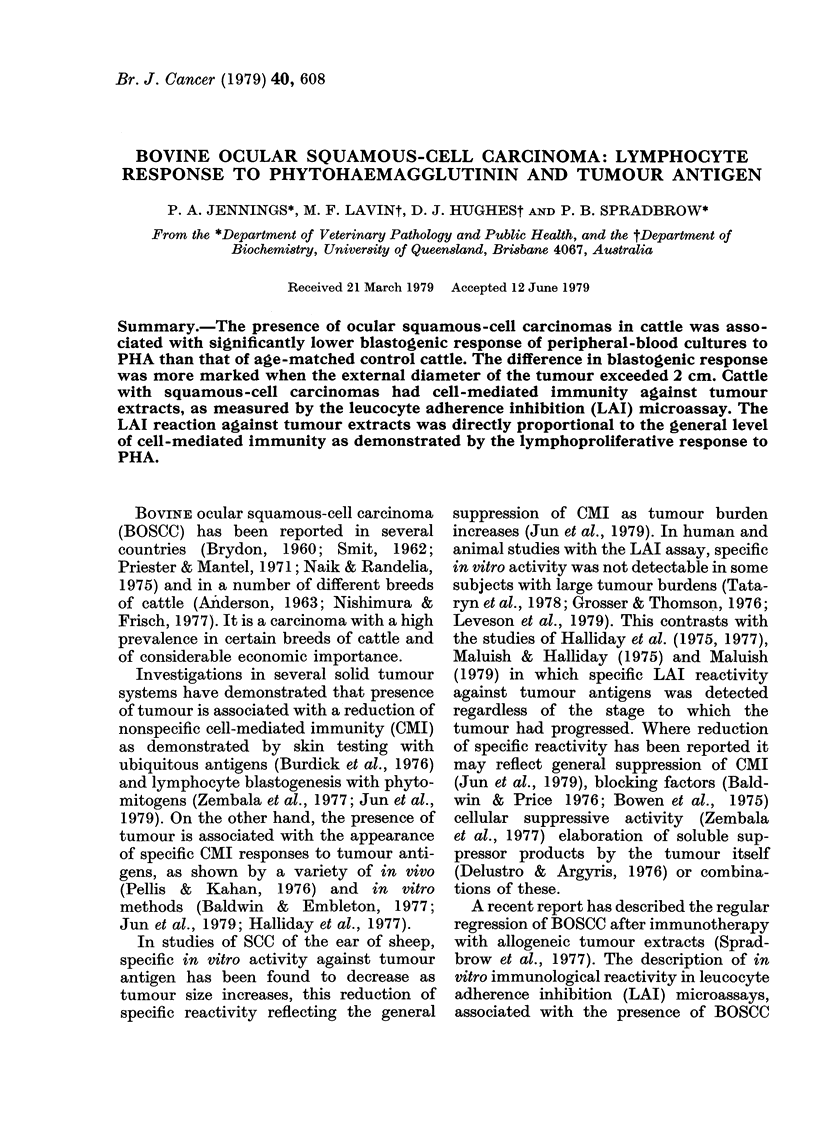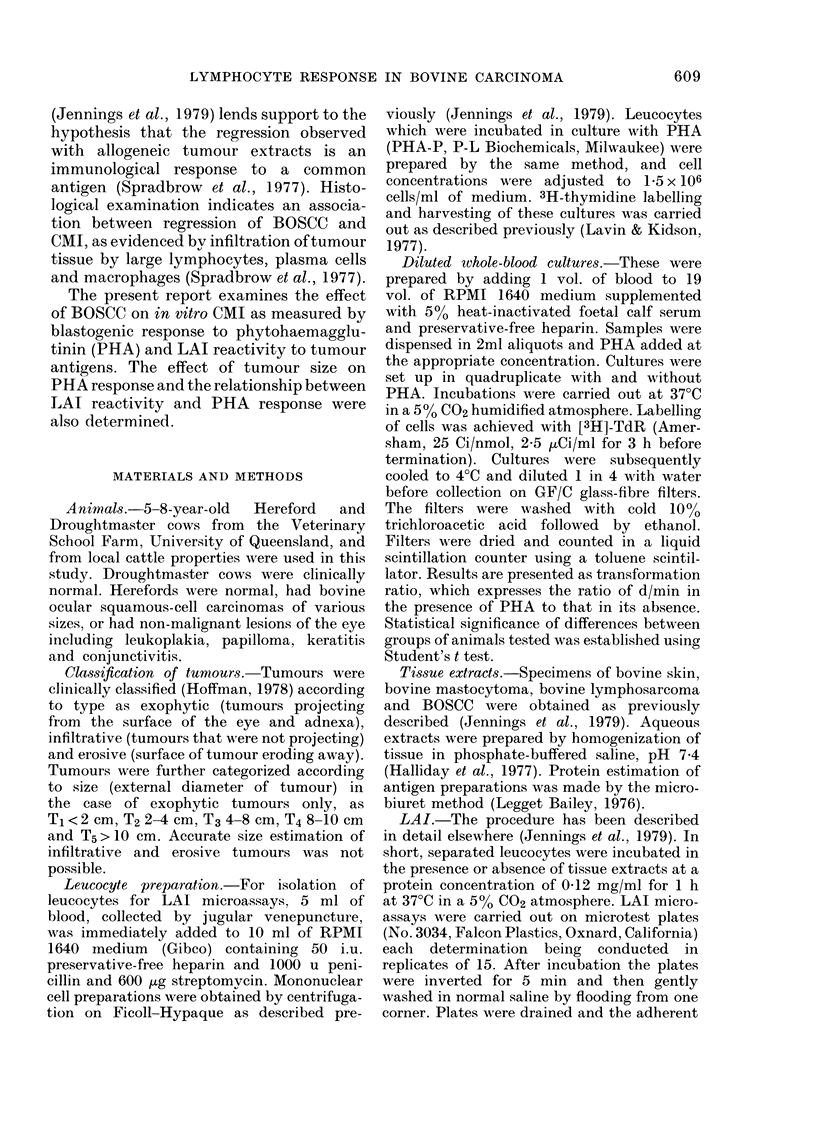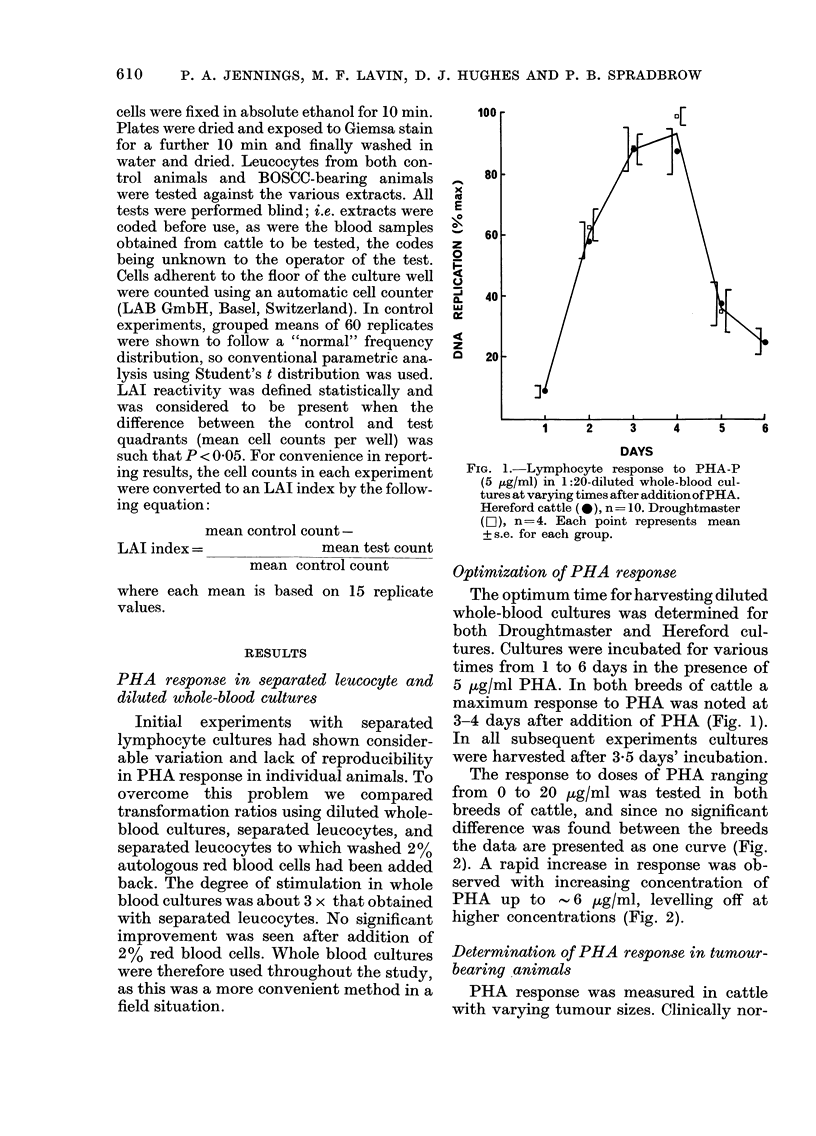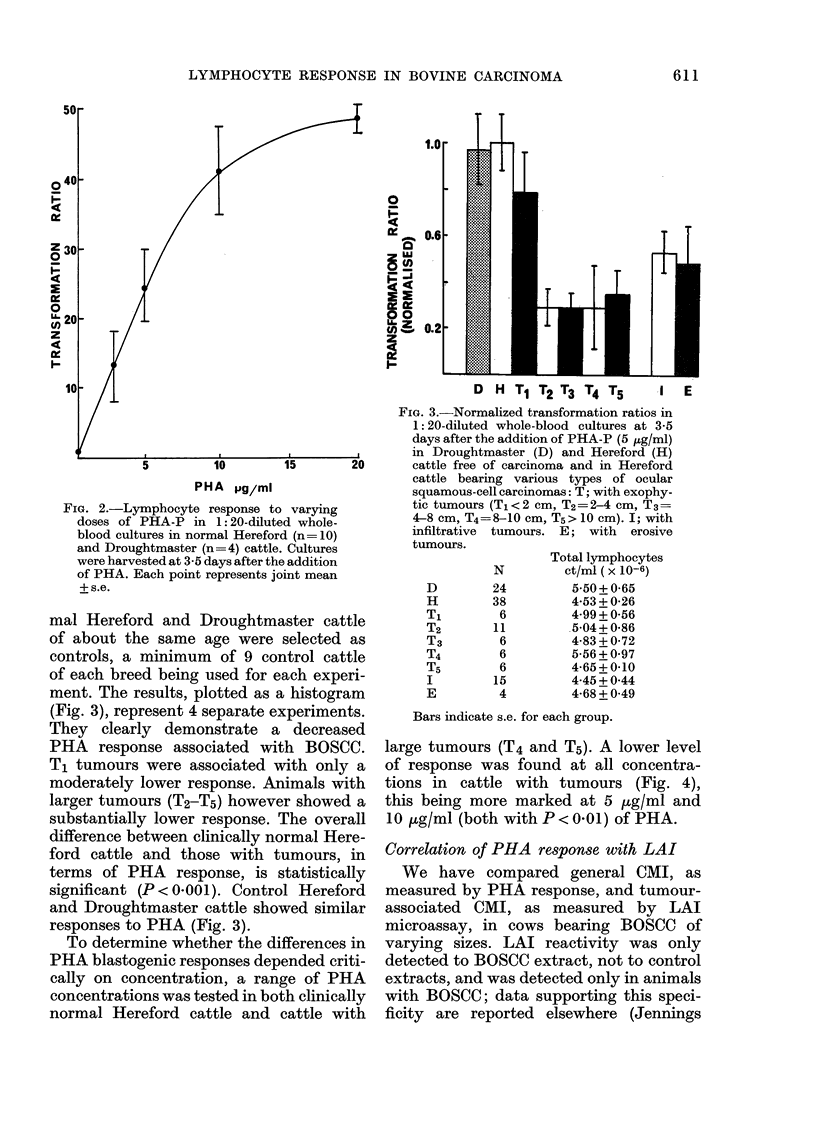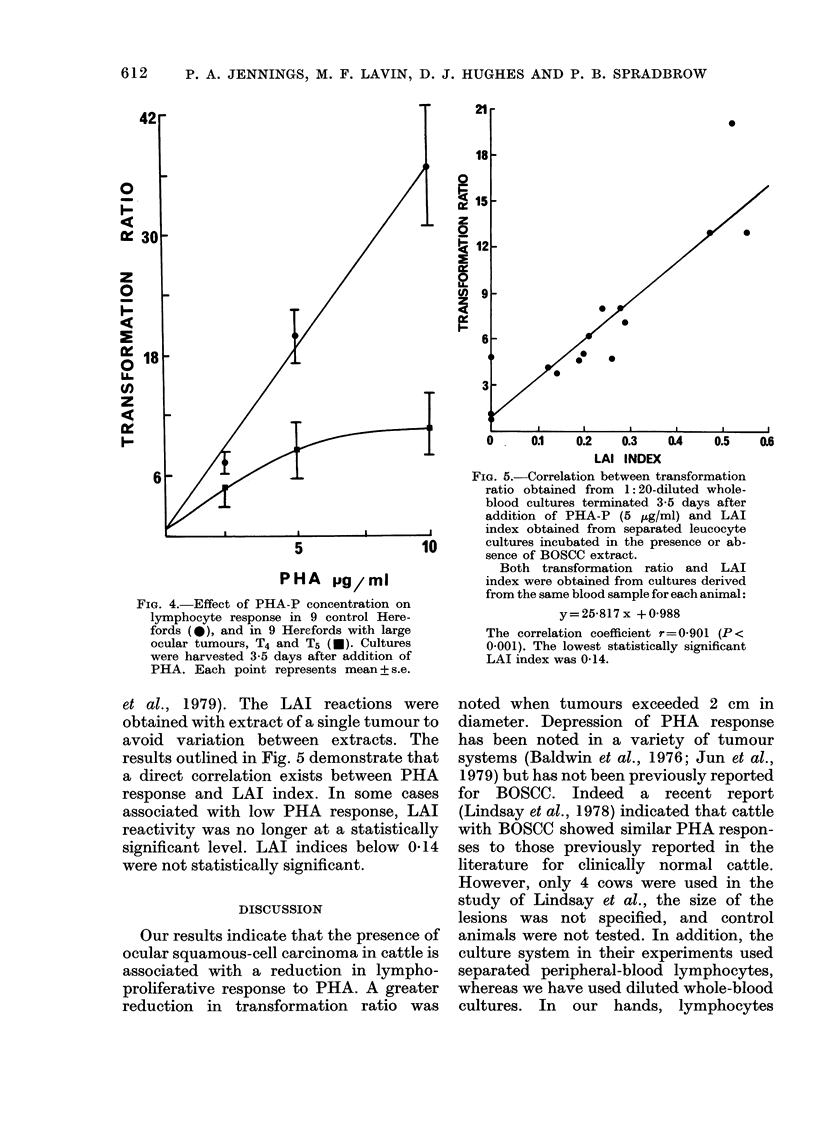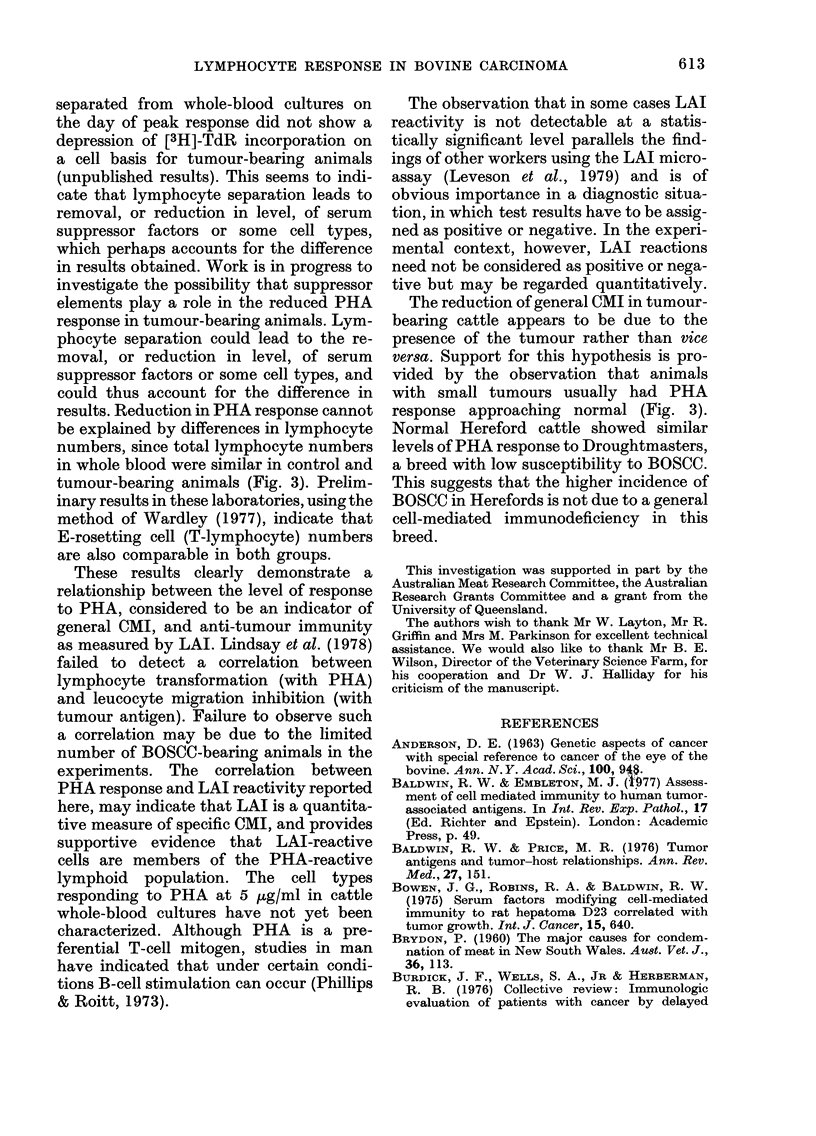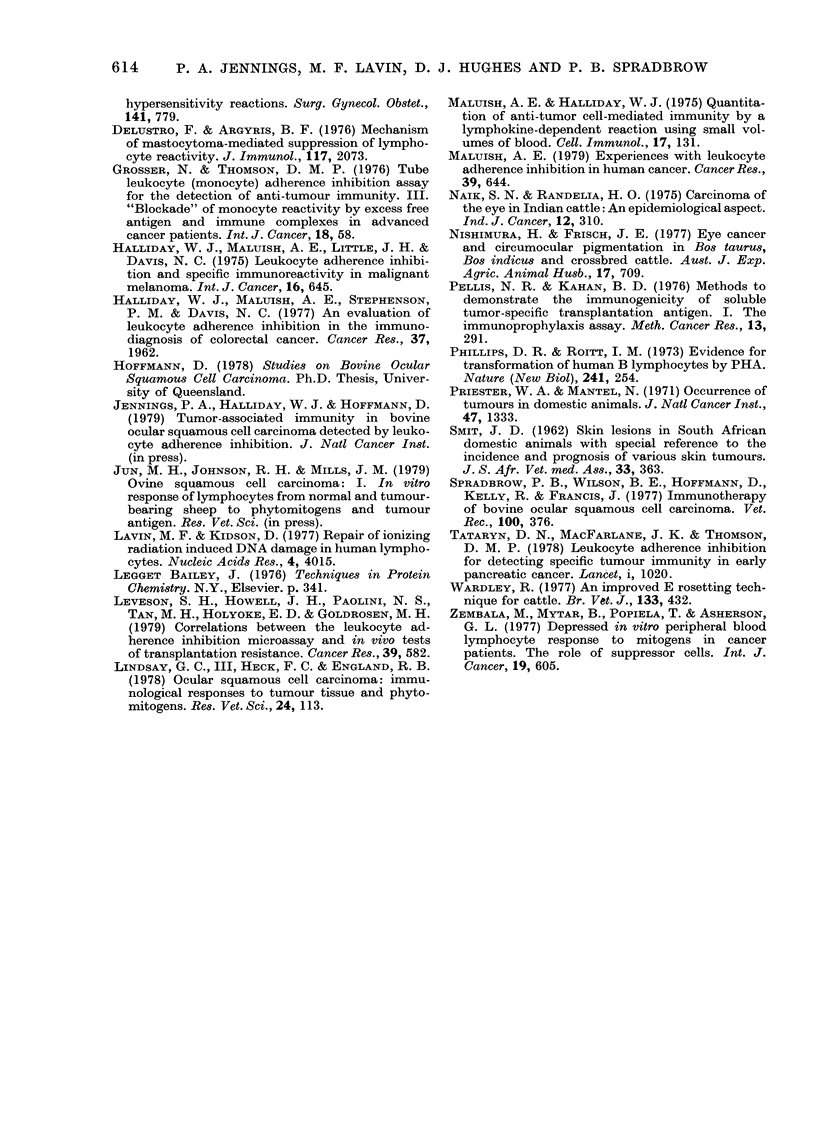# Bovine ocular squamous-cell carcinoma: lymphocyte response to phytohaemagglutinin and tumour antigen.

**DOI:** 10.1038/bjc.1979.226

**Published:** 1979-10

**Authors:** P. A. Jennings, M. F. Lavin, D. J. Hughes, P. B. Spradbrow

## Abstract

The presence of ocular squamous-cell carcinomas in cattle was associated with significantly lower blastogenic response of peripheral-blood cultures to PHA than that of age-matched control cattle. The difference in blastogenic response was more marked when the external diameter of the tumour exceeded 2 cm. Cattle with aquamous-cell carcinomas had cell-mediated immunity against tumour extracts, as measured by the leucocyte adherence inhibition (LAI) microassay. The LAI reaction against tumour extracts was directly proportional to the general level of cell-mediated immunity as demonstrated by the lymphoproliferative response to PHA.


Br. J. Cancer (1979) 40, 608

### BOVINE OCULAR SQUAMOUS-CELL CARCINOMA: LYMPHOCYTE RESPONSE TO PHYTOHAEMAGGLUTININ AND TUMOUR ANTIGEN

P. A. JENNINGS*, M. F. LAVIN†, D. J. HUGHES† AND P. B. SPRADBROW*

*From the *Department of Veterinary Pathology and Public Health, and the †Department of Biochemistry, University of Queensland, Brisbane 4067, Australia*

Received 21 March 1979 Accepted 12 June 1979

**Summary.—The presence of ocular squamous-cell carcinomas in cattle was associated with significantly lower blastogenic response of peripheral-blood cultures to PHA than that of age-matched control cattle. The difference in blastogenic response was more marked when the external diameter of the tumour exceeded 2 cm. Cattle with squamous-cell carcinomas had cell-mediated immunity against tumour extracts, as measured by the leucocyte adherence inhibition (LAI) microassay. The LAI reaction against tumour extracts was directly proportional to the general level of cell-mediated immunity as demonstrated by the lymphoproliferative response to PHA.**

Bovine ocular squamous-cell carcinoma (BOSCC) has been reported in several countries (Brydon, 1960; Smit, 1962; Priester & Mantel, 1971; Naik & Randelia, 1975) and in a number of different breeds of cattle (Anderson, 1963; Nishimura & Frisch, 1977). It is a carcinoma with a high prevalence in certain breeds of cattle and of considerable economic importance.

Investigations in several solid tumour systems have demonstrated that presence of tumour is associated with a reduction of nonspecific cell-mediated immunity (CMI) as demonstrated by skin testing with ubiquitous antigens (Burdick *et al.*, 1976) and lymphocyte blastogenesis with phytomitogens (Zembala *et al.*, 1977; Jun *et al.*, 1979). On the other hand, the presence of tumour is associated with the appearance of specific CMI responses to tumour antigens, as shown by a variety of *in vivo* (Pellis & Kahan, 1976) and *in vitro* methods (Baldwin & Embleton, 1977; Jun *et al.*, 1979; Halliday *et al.*, 1977).

In studies of SCC of the ear of sheep, specific *in vitro* activity against tumour antigen has been found to decrease as tumour size increases, this reduction of specific reactivity reflecting the general suppression of CMI as tumour burden increases (Jun *et al.*, 1979). In human and animal studies with the LAI assay, specific *in vitro* activity was not detectable in some subjects with large tumour burdens (Tataryn *et al.*, 1978; Grosser & Thomson, 1976; Leveson *et al.*, 1979). This contrasts with the studies of Halliday *et al.* (1975, 1977), Maluish & Halliday (1975) and Maluish (1979) in which specific LAI reactivity against tumour antigens was detected regardless of the stage to which the tumour had progressed. Where reduction of specific reactivity has been reported it may reflect general suppression of CMI (Jun *et al.*, 1979), blocking factors (Baldwin & Price 1976; Bowen *et al.*, 1975) cellular suppressive activity (Zembala *et al.*, 1977) elaboration of soluble suppressor products by the tumour itself (Delustro & Argyris, 1976) or combinations of these.

A recent report has described the regular regression of BOSCC after immunotherapy with allogeneic tumour extracts (Spradbrow *et al.*, 1977). The description of *in vitro* immunological reactivity in leucocyte adherence inhibition (LAI) microassays, associated with the presence of BOSCC

(Jennings *et al.*, 1979) lends support to the hypothesis that the regression observed with allogeneic tumour extracts is an immunological response to a common antigen (Spradbrow *et al.*, 1977). Histological examination indicates an association between regression of BOSCC and CMI, as evidenced by infiltration of tumour tissue by large lymphocytes, plasma cells and macrophages (Spradbrow *et al.*, 1977).

The present report examines the effect of BOSCC on *in vitro* CMI as measured by blastogenic response to phytohaemagglutinin (PHA) and LAI reactivity to tumour antigens. The effect of tumour size on PHA response and the relationship between LAI reactivity and PHA response were also determined.

### MATERIALS AND METHODS

*Animals.*—5–8-year-old Hereford and Droughtmaster cows from the Veterinary School Farm, University of Queensland, and from local cattle properties were used in this study. Droughtmaster cows were clinically normal. Herefords were normal, had bovine ocular squamous-cell carcinomas of various sizes, or had non-malignant lesions of the eye including leukoplakia, papilloma, keratitis and conjunctivitis.

*Classification of tumours.*—Tumours were clinically classified (Hoffman, 1978) according to type as exophytic (tumours projecting from the surface of the eye and adnexa), infiltrative (tumours that were not projecting) and erosive (surface of tumour eroding away). Tumours were further categorized according to size (external diameter of tumour) in the case of exophytic tumours only, as $T_1 < 2$ cm, $T_2$ 2–4 cm, $T_3$ 4–8 cm, $T_4$ 8–10 cm and $T_5 > 10$ cm. Accurate size estimation of infiltrative and erosive tumours was not possible.

*Leucocyte preparation.*—For isolation of leucocytes for LAI microassays, 5 ml of blood, collected by jugular venepuncture, was immediately added to 10 ml of RPMI 1640 medium (Gibco) containing 50 i.u. preservative-free heparin and 1000 u penicillin and 600 $\mu$g streptomycin. Mononuclear cell preparations were obtained by centrifugation on Ficoll–Hypaque as described previously (Jennings *et al.*, 1979). Leucocytes which were incubated in culture with PHA (PHA-P, P-L Biochemicals, Milwaukee) were prepared by the same method, and cell concentrations were adjusted to $1{\cdot}5 \times 10^6$ cells/ml of medium. $^3$H-thymidine labelling and harvesting of these cultures was carried out as described previously (Lavin & Kidson, 1977).

*Diluted whole-blood cultures.*—These were prepared by adding 1 vol. of blood to 19 vol. of RPMI 1640 medium supplemented with 5% heat-inactivated foetal calf serum and preservative-free heparin. Samples were dispensed in 2ml aliquots and PHA added at the appropriate concentration. Cultures were set up in quadruplicate with and without PHA. Incubations were carried out at 37°C in a 5% $CO_2$ humidified atmosphere. Labelling of cells was achieved with [$^3$H]-TdR (Amersham, 25 Ci/nmol, 2·5 $\mu$Ci/ml for 3 h before termination). Cultures were subsequently cooled to 4°C and diluted 1 in 4 with water before collection on GF/C glass-fibre filters. The filters were washed with cold 10% trichloroacetic acid followed by ethanol. Filters were dried and counted in a liquid scintillation counter using a toluene scintillator. Results are presented as transformation ratio, which expresses the ratio of d/min in the presence of PHA to that in its absence. Statistical significance of differences between groups of animals tested was established using Student's *t* test.

*Tissue extracts.*—Specimens of bovine skin, bovine mastocytoma, bovine lymphosarcoma and BOSCC were obtained as previously described (Jennings *et al.*, 1979). Aqueous extracts were prepared by homogenization of tissue in phosphate-buffered saline, pH 7·4 (Halliday *et al.*, 1977). Protein estimation of antigen preparations was made by the microbiuret method (Legget Bailey, 1976).

*LAI.*—The procedure has been described in detail elsewhere (Jennings *et al.*, 1979). In short, separated leucocytes were incubated in the presence or absence of tissue extracts at a protein concentration of 0·12 mg/ml for 1 h at 37°C in a 5% $CO_2$ atmosphere. LAI microassays were carried out on microtest plates (No. 3034, Falcon Plastics, Oxnard, California) each determination being conducted in replicates of 15. After incubation the plates were inverted for 5 min and then gently washed in normal saline by flooding from one corner. Plates were drained and the adherent cells were fixed in absolute ethanol for 10 min. Plates were dried and exposed to Giemsa stain for a further 10 min and finally washed in water and dried. Leucocytes from both control animals and BOSCC-bearing animals were tested against the various extracts. All tests were performed blind; *i.e.* extracts were coded before use, as were the blood samples obtained from cattle to be tested, the codes being unknown to the operator of the test. Cells adherent to the floor of the culture well were counted using an automatic cell counter (LAB GmbH, Basel, Switzerland). In control experiments, grouped means of 60 replicates were shown to follow a "normal" frequency distribution, so conventional parametric analysis using Student's $t$ distribution was used. LAI reactivity was defined statistically and was considered to be present when the difference between the control and test quadrants (mean cell counts per well) was such that $P < 0{\cdot}05$. For convenience in reporting results, the cell counts in each experiment were converted to an LAI index by the following equation:

$$\text{LAI index} = \frac{\text{mean control count} - \text{mean test count}}{\text{mean control count}}$$

where each mean is based on 15 replicate values.

### RESULTS

### *PHA response in separated leucocyte and diluted whole-blood cultures*

Initial experiments with separated lymphocyte cultures had shown considerable variation and lack of reproducibility in PHA response in individual animals. To overcome this problem we compared transformation ratios using diluted whole-blood cultures, separated leucocytes, and separated leucocytes to which washed 2% autologous red blood cells had been added back. The degree of stimulation in whole blood cultures was about 3 × that obtained with separated leucocytes. No significant improvement was seen after addition of 2% red blood cells. Whole blood cultures were therefore used throughout the study, as this was a more convenient method in a field situation.

FIG. 1.—Lymphocyte response to PHA-P (5 μg/ml) in 1:20-diluted whole-blood cultures at varying times after addition of PHA. Hereford cattle (●), n = 10. Droughtmaster (□), n = 4. Each point represents mean ± s.e. for each group.

### *Optimization of PHA response*

The optimum time for harvesting diluted whole-blood cultures was determined for both Droughtmaster and Hereford cultures. Cultures were incubated for various times from 1 to 6 days in the presence of 5 μg/ml PHA. In both breeds of cattle a maximum response to PHA was noted at 3–4 days after addition of PHA (Fig. 1). In all subsequent experiments cultures were harvested after 3·5 days' incubation.

The response to doses of PHA ranging from 0 to 20 μg/ml was tested in both breeds of cattle, and since no significant difference was found between the breeds the data are presented as one curve (Fig. 2). A rapid increase in response was observed with increasing concentration of PHA up to ~6 μg/ml, levelling off at higher concentrations (Fig. 2).

### *Determination of PHA response in tumour-bearing animals*

PHA response was measured in cattle with varying tumour sizes. Clinically nor-

FIG. 2.—Lymphocyte response to varying doses of PHA-P in 1:20-diluted whole-blood cultures in normal Hereford (n = 10) and Droughtmaster (n = 4) cattle. Cultures were harvested at 3·5 days after the addition of PHA. Each point represents joint mean ± s.e.

FIG. 3.—Normalized transformation ratios in 1:20-diluted whole-blood cultures at 3·5 days after the addition of PHA-P (5 µg/ml) in Droughtmaster (D) and Hereford (H) cattle free of carcinoma and in Hereford cattle bearing various types of ocular squamous-cell carcinomas: T; with exophytic tumours ($T_1 < 2$ cm, $T_2 = 2$–4 cm, $T_3 =$ 4–8 cm, $T_4 = 8$–10 cm, $T_5 > 10$ cm). I; with infiltrative tumours. E; with erosive tumours.

| | N | Total lymphocytes ct/ml ($\times 10^{-6}$) |
|---|---|---|
| D | 24 | 5·50 ± 0·65 |
| H | 38 | 4·53 ± 0·26 |
| $T_1$ | 6 | 4·99 ± 0·56 |
| $T_2$ | 11 | 5·04 ± 0·86 |
| $T_3$ | 6 | 4·83 ± 0·72 |
| $T_4$ | 6 | 5·56 ± 0·97 |
| $T_5$ | 6 | 4·65 ± 0·10 |
| I | 15 | 4·45 ± 0·44 |
| E | 4 | 4·68 ± 0·49 |

Bars indicate s.e. for each group.

mal Hereford and Droughtmaster cattle of about the same age were selected as controls, a minimum of 9 control cattle of each breed being used for each experiment. The results, plotted as a histogram (Fig. 3), represent 4 separate experiments. They clearly demonstrate a decreased PHA response associated with BOSCC. $T_1$ tumours were associated with only a moderately lower response. Animals with larger tumours ($T_2$–$T_5$) however showed a substantially lower response. The overall difference between clinically normal Hereford cattle and those with tumours, in terms of PHA response, is statistically significant ($P < 0{\cdot}001$). Control Hereford and Droughtmaster cattle showed similar responses to PHA (Fig. 3).

To determine whether the differences in PHA blastogenic responses depended critically on concentration, a range of PHA concentrations was tested in both clinically normal Hereford cattle and cattle with large tumours ($T_4$ and $T_5$). A lower level of response was found at all concentrations in cattle with tumours (Fig. 4), this being more marked at 5 µg/ml and 10 µg/ml (both with $P < 0{\cdot}01$) of PHA.

*Correlation of PHA response with LAI*

We have compared general CMI, as measured by PHA response, and tumour-associated CMI, as measured by LAI microassay, in cows bearing BOSCC of varying sizes. LAI reactivity was only detected to BOSCC extract, not to control extracts, and was detected only in animals with BOSCC; data supporting this specificity are reported elsewhere (Jennings

FIG. 4.—Effect of PHA-P concentration on lymphocyte response in 9 control Herefords (●), and in 9 Herefords with large ocular tumours, $T_4$ and $T_5$ (■). Cultures were harvested 3·5 days after addition of PHA. Each point represents mean ± s.e.

FIG. 5.—Correlation between transformation ratio obtained from 1:20-diluted whole-blood cultures terminated 3·5 days after addition of PHA-P (5 $\mu$g/ml) and LAI index obtained from separated leucocyte cultures incubated in the presence or absence of BOSCC extract.

Both transformation ratio and LAI index were obtained from cultures derived from the same blood sample for each animal:

$$y = 25{\cdot}817\,x + 0{\cdot}988$$

The correlation coefficient $r = 0{\cdot}901$ ($P < 0{\cdot}001$). The lowest statistically significant LAI index was 0·14.

*et al.*, 1979). The LAI reactions were obtained with extract of a single tumour to avoid variation between extracts. The results outlined in Fig. 5 demonstrate that a direct correlation exists between PHA response and LAI index. In some cases associated with low PHA response, LAI reactivity was no longer at a statistically significant level. LAI indices below 0·14 were not statistically significant.

### DISCUSSION

Our results indicate that the presence of ocular squamous-cell carcinoma in cattle is associated with a reduction in lymphoproliferative response to PHA. A greater reduction in transformation ratio was noted when tumours exceeded 2 cm in diameter. Depression of PHA response has been noted in a variety of tumour systems (Baldwin *et al.*, 1976; Jun *et al.*, 1979) but has not been previously reported for BOSCC. Indeed a recent report (Lindsay *et al.*, 1978) indicated that cattle with BOSCC showed similar PHA responses to those previously reported in the literature for clinically normal cattle. However, only 4 cows were used in the study of Lindsay *et al.*, the size of the lesions was not specified, and control animals were not tested. In addition, the culture system in their experiments used separated peripheral-blood lymphocytes, whereas we have used diluted whole-blood cultures. In our hands, lymphocytes separated from whole-blood cultures on the day of peak response did not show a depression of [$^3$H]-TdR incorporation on a cell basis for tumour-bearing animals (unpublished results). This seems to indicate that lymphocyte separation leads to removal, or reduction in level, of serum suppressor factors or some cell types, which perhaps accounts for the difference in results obtained. Work is in progress to investigate the possibility that suppressor elements play a role in the reduced PHA response in tumour-bearing animals. Lymphocyte separation could lead to the removal, or reduction in level, of serum suppressor factors or some cell types, and could thus account for the difference in results. Reduction in PHA response cannot be explained by differences in lymphocyte numbers, since total lymphocyte numbers in whole blood were similar in control and tumour-bearing animals (Fig. 3). Preliminary results in these laboratories, using the method of Wardley (1977), indicate that E-rosetting cell (T-lymphocyte) numbers are also comparable in both groups.

These results clearly demonstrate a relationship between the level of response to PHA, considered to be an indicator of general CMI, and anti-tumour immunity as measured by LAI. Lindsay *et al.* (1978) failed to detect a correlation between lymphocyte transformation (with PHA) and leucocyte migration inhibition (with tumour antigen). Failure to observe such a correlation may be due to the limited number of BOSCC-bearing animals in the experiments. The correlation between PHA response and LAI reactivity reported here, may indicate that LAI is a quantitative measure of specific CMI, and provides supportive evidence that LAI-reactive cells are members of the PHA-reactive lymphoid population. The cell types responding to PHA at 5 $\mu$g/ml in cattle whole-blood cultures have not yet been characterized. Although PHA is a preferential T-cell mitogen, studies in man have indicated that under certain conditions B-cell stimulation can occur (Phillips & Roitt, 1973).

The observation that in some cases LAI reactivity is not detectable at a statistically significant level parallels the findings of other workers using the LAI microassay (Leveson *et al.*, 1979) and is of obvious importance in a diagnostic situation, in which test results have to be assigned as positive or negative. In the experimental context, however, LAI reactions need not be considered as positive or negative but may be regarded quantitatively.

The reduction of general CMI in tumour-bearing cattle appears to be due to the presence of the tumour rather than *vice versa*. Support for this hypothesis is provided by the observation that animals with small tumours usually had PHA response approaching normal (Fig. 3). Normal Hereford cattle showed similar levels of PHA response to Droughtmasters, a breed with low susceptibility to BOSCC. This suggests that the higher incidence of BOSCC in Herefords is not due to a general cell-mediated immunodeficiency in this breed.

This investigation was supported in part by the Australian Meat Research Committee, the Australian Research Grants Committee and a grant from the University of Queensland.

The authors wish to thank Mr W. Layton, Mr R. Griffin and Mrs M. Parkinson for excellent technical assistance. We would also like to thank Mr B. E. Wilson, Director of the Veterinary Science Farm, for his cooperation and Dr W. J. Halliday for his criticism of the manuscript.

### REFERENCES

ANDERSON, D. E. (1963) Genetic aspects of cancer with special reference to cancer of the eye of the bovine. *Ann. N.Y. Acad. Sci.*, **100**, 948.

BALDWIN, R. W. & EMBLETON, M. J. (1977) Assessment of cell mediated immunity to human tumor-associated antigens. In *Int. Rev. Exp. Pathol.*, **17** (Ed. Richter and Epstein). London: Academic Press, p. 49.

BALDWIN, R. W. & PRICE, M. R. (1976) Tumor antigens and tumor–host relationships. *Ann. Rev. Med.*, **27**, 151.

BOWEN, J. G., ROBINS, R. A. & BALDWIN, R. W. (1975) Serum factors modifying cell-mediated immunity to rat hepatoma D23 correlated with tumor growth. *Int. J. Cancer*, **15**, 640.

BRYDON, P. (1960) The major causes for condemnation of meat in New South Wales. *Aust. Vet. J.*, **36**, 113.

BURDICK, J. F., WELLS, S. A., JR & HERBERMAN, R. B. (1976) Collective review: Immunologic evaluation of patients with cancer by delayed hypersensitivity reactions. *Surg. Gynecol. Obstet.*, **141**, 779.

DELUSTRO, F. & ARGYRIS, B. F. (1976) Mechanism of mastocytoma-mediated suppression of lymphocyte reactivity. *J. Immunol.*, **117**, 2073.

GROSSER, N. & THOMSON, D. M. P. (1976) Tube leukocyte (monocyte) adherence inhibition assay for the detection of anti-tumour immunity. III. "Blockade" of monocyte reactivity by excess free antigen and immune complexes in advanced cancer patients. *Int. J. Cancer*, **18**, 58.

HALLIDAY, W. J., MALUISH, A. E., LITTLE, J. H. & DAVIS, N. C. (1975) Leukocyte adherence inhibition and specific immunoreactivity in malignant melanoma. *Int. J. Cancer*, **16**, 645.

HALLIDAY, W. J., MALUISH, A. E., STEPHENSON, P. M. & DAVIS, N. C. (1977) An evaluation of leukocyte adherence inhibition in the immunodiagnosis of colorectal cancer. *Cancer Res.*, **37**, 1962.

HOFFMANN, D. (1978) *Studies on Bovine Ocular Squamous Cell Carcinoma*. Ph.D. Thesis, University of Queensland.

JENNINGS, P. A., HALLIDAY, W. J. & HOFFMANN, D. (1979) Tumor-associated immunity in bovine ocular squamous cell carcinoma detected by leukocyte adherence inhibition. *J. Natl Cancer Inst.* (in press).

JUN, M. H., JOHNSON, R. H. & MILLS, J. M. (1979) Ovine squamous cell carcinoma: I. *In vitro* response of lymphocytes from normal and tumour-bearing sheep to phytomitogens and tumour antigen. *Res. Vet. Sci.* (in press).

LAVIN, M. F. & KIDSON, D. (1977) Repair of ionizing radiation induced DNA damage in human lymphocytes. *Nucleic Acids Res.*, **4**, 4015.

LEGGET BAILEY, J. (1976) *Techniques in Protein Chemistry*. N.Y., Elsevier. p. 341.

LEVESON, S. H., HOWELL, J. H., PAOLINI, N. S., TAN, M. H., HOLYOKE, E. D. & GOLDROSEN, M. H. (1979) Correlations between the leukocyte adherence inhibition microassay and *in vivo* tests of transplantation resistance. *Cancer Res.*, **39**, 582.

LINDSAY, G. C., III, HECK, F. C. & ENGLAND, R. B. (1978) Ocular squamous cell carcinoma: immunological responses to tumour tissue and phytomitogens. *Res. Vet. Sci.*, **24**, 113.

MALUISH, A. E. & HALLIDAY, W. J. (1975) Quantitation of anti-tumor cell-mediated immunity by a lymphokine-dependent reaction using small volumes of blood. *Cell. Immunol.*, **17**, 131.

MALUISH, A. E. (1979) Experiences with leukocyte adherence inhibition in human cancer. *Cancer Res.*, **39**, 644.

NAIK, S. N. & RANDELIA, H. O. (1975) Carcinoma of the eye in Indian cattle: An epidemiological aspect. *Ind. J. Cancer*, **12**, 310.

NISHIMURA, H. & FRISCH, J. E. (1977) Eye cancer and circumocular pigmentation in *Bos taurus*, *Bos indicus* and crossbred cattle. *Aust. J. Exp. Agric. Animal Husb.*, **17**, 709.

PELLIS, N. R. & KAHAN, B. D. (1976) Methods to demonstrate the immunogenicity of soluble tumor-specific transplantation antigen. I. The immunoprophylaxis assay. *Meth. Cancer Res.*, **13**, 291.

PHILLIPS, D. R. & ROITT, I. M. (1973) Evidence for transformation of human B lymphocytes by PHA. *Nature* (*New Biol*), **241**, 254.

PRIESTER, W. A. & MANTEL, N. (1971) Occurrence of tumours in domestic animals. *J. Natl Cancer Inst.*, **47**, 1333.

SMIT, J. D. (1962) Skin lesions in South African domestic animals with special reference to the incidence and prognosis of various skin tumours. *J. S. Afr. Vet. med. Ass.*, **33**, 363.

SPRADBROW, P. B., WILSON, B. E., HOFFMANN, D., KELLY, R. & FRANCIS, J. (1977) Immunotherapy of bovine ocular squamous cell carcinoma. *Vet. Rec.*, **100**, 376.

TATARYN, D. N., MACFARLANE, J. K. & THOMSON, D. M. P. (1978) Leukocyte adherence inhibition for detecting specific tumour immunity in early pancreatic cancer. *Lancet*, i, 1020.

WARDLEY, R. (1977) An improved E rosetting technique for cattle. *Br. Vet. J.*, **133**, 432.

ZEMBALA, M., MYTAR, B., POPIELA, T. & ASHERSON, G. L. (1977) Depressed *in vitro* peripheral blood lymphocyte response to mitogens in cancer patients. The role of suppressor cells. *Int. J. Cancer*, **19**, 605.